# Effects of Gene × Attachment Interaction on Adolescents’ Emotion Regulation and Aggressive Hostile Behavior Towards their Mothers during a Computer Game

**DOI:** 10.3389/fnhum.2016.00254

**Published:** 2016-06-14

**Authors:** Peter Zimmermann, Gottfried Spangler

**Affiliations:** ^1^Department of Developmental Psychology, University of WuppertalWuppertal, Germany; ^2^Department of Psychology, Friedrich-Alexander-University of Erlangen-NurembergErlangen, Germany

**Keywords:** 5-HTTLPR, attachment, emotionality, emotion regulation, adolescence, autonomy, aggression, gene × environment interaction

## Abstract

Adolescence is a time of increased emotionality and major changes in emotion regulation often elicited in autonomy-relevant situations. Both genetic as well as social factors may lead to inter-individual differences in emotional processes in adolescence. We investigated whether both 5-HTTLPR and attachment security influence adolescents’ observed emotionality, emotional dysregulation, and their aggressive hostile autonomy while interacting with their mothers. Eighty-eight adolescents at age 12 were observed in interaction with their mothers during a standardized, emotion eliciting computer game task. They were genotyped for the 5-HTTLPR, a repeat polymorphism in the promoter region of the serotonin transporter gene. Concurrent attachment quality was assessed by the Late Childhood Attachment Interview (LCAI). Results revealed a significant gene × attachment effect showing that ss/sl carriers of 5-HTTLPR show increased emotional dysregulation and aggressive hostile autonomy towards their mothers. The results of the study suggest that secure attachment in adolescence moderates the genetically based higher tendency for emotional dysregulation and aggressive reactions to restrictions of autonomy during emotional social interactions with their mothers.

## Introduction

### Adolescence, Autonomy, and Emotionality

Adolescence is characterized by increased emotionality and daily mood fluctuations especially in early adolescence (Larson et al., 2002; Maciejewski et al., 2015). This may be due to hormonal changes around puberty, brain development, cognitive, and social changes and stressors (Laursen, 1995; Forbes and Dahl, 2010; Somerville et al., 2010). Parallel, the elicitors of emotions change from childhood to adolescence. Social evaluation, especially by peers becomes a major elicitor of fear (Westenberg et al., 2004; Guyer et al., 2014). Autonomy restrictions, especially by parents, become major triggers (Laursen, 1995; Oudekerk et al., 2015) for impulsive anger, aggressive quarrel or sadness that also affects psychophysiological regulation during adolescent-parent interactions (Cook et al., 2015). Autonomy, the need for developing own goals and trying to reach them is a stage-salient issue for adolescents. Therefore, adolescents experience intense negative emotions when they are confronted with goal blocking in this domain.

These developmental changes and stage-salient issues challenge the adolescents’ emotion regulation capacities but also that of their caregivers. Emotion regulation during adolescence becomes increasingly more effective and adaptive (Silk et al., 2003; Zimmermann and Iwanski, 2014). However, inter-individual differences in emotion regulation and emotional reactions to autonomy restrictions exist and partly are explained by genetic variations and social factors.

### Genetic Effects on Emotionality, Emotion Regulation, and Aggression

Research on the associations between specific candidate genes and emotionality or emotion regulation has provided some evidence for the relevance of genetic polymorphisms of the serotonin system and the dopamine system in both domains (Munafò et al., 2008; Canli et al., 2009; Hawn et al., 2015). Canli and Lesch (2007) specifically emphasized the influence of the serotonin transporter 5-HTT gene on emotionality and emotion regulation. The 5-HTT polymorphism leads to differences in serotonin neurotransmission with the short (S) allele variants displaying significantly less 5-HTT binding in the brain than the homozygous long (L) variant (Murphy and Lesch, 2008). S-allele carrier show impaired functional integration of cortico-limbic connectivity and poorer inhibitory regulation via the prefrontal cortex that leads to an increased reactivity of the amygdala to emotionally provocative stimuli (Hariri and Holmes, 2006). In general, they seem to exhibit a higher emotional reactivity (i.e., a lower threshold to emotional stimuli and increased arousal) and a biased emotional information processing associated with an even higher baseline amygdala activity (Canli and Lesch, 2007). Fernàndez-Castillo and Cormand (2016) reported in their review, a clear link between the short allele variant and increased impulsivity and aggressiveness in humans. This might specifically be the case for impulsive aggression (Hennig et al., 2005). In a similar vein, Cyders and Smith (2008) proposed the idea that genetic polymorphisms affecting the serotonin and the dopamine system explain impulsive and rash reactions, especially in emotionally arousing situations. In summary, S-allele carriers seem to represent an emotional phenotype characterized by increased emotional reactivity and impaired capacity to regulate emotions, which finds its expression in impulsive aggressive actions on the behavioral level.

However, single genetic polymorphisms only explain parts of the variability of human traits or habitual patterns of emotionality and emotion regulation. Therefore, the interaction with other genetic variations and with environmental factors often moderates the effects of single genes on behavior. From a developmental perspective, a transactional model of genetic disposition, environmental factors, epigenetic changes, and the active role of the individual in selecting and shaping the environment is a more appropriate approach in understanding the development of emotion regulation (Sameroff, 2010). Thus, especially in adolescence possible effects of the individual genetic dispositions on eliciting specific responses in the social environment (e.g., their mothers) need to be considered. This includes, whether adolescent’s genotype explains the eliciting of parenting (Oppenheimer et al., 2013) or whether mother’s genotype explains variation in her sensitivity towards the adolescent in dyadic interactions (Bakermans-Kranenburg and van IJzendoorn, 2008), so that maternal genotype indirectly would explain differences in the adolescents’ emotion regulation and autonomy.

Within the life span, emotion regulation in infancy starts with dominantly social emotion regulation where the infant depends on the caregiver’s comfort and support. With increasing age, individual emotion regulation becomes more prominent and new emotion regulation strategies are integrated into the individual self-regulation repertoire (Zimmermann and Thompson, 2014). However, social and cultural factors moderate this process (Cole, 2014) and one of these central moderating factors is attachment.

### Attachment and Emotion Regulation

The attachment system is a biologically based security regulation system that is activated by existential threat or intense negative emotions (Bowlby, 1980). Although initially developed in infancy, attachment can still be elicited and has regulatory functions during middle childhood, adolescence, and adulthood (Allen and Land, 2008; Grossmann et al., 2008; Bosmans and Kerns, 2015). However, in contrast to childhood, adolescents less often express their attachment needs by seeking proximity in time of distress (although they still do). More often, they show psychological proximity seeking by means of emotional communication with the caregiver when needed. By that, adolescents balance the stage-salient issue of autonomy development with maintaining attachment (Kobak et al., 1993; Allen and Land, 2008; Becker-Stoll et al., 2008). However, the importance of adolescents’ verbal emotional communication with their caregiver for emotion regulation is obvious in the reduced adrenocortical activity in adolescents who have been able to talk to their mothers after having been stressed in the Trier Social Stress Test (Seltzer et al., 2012).

Attachment influences several domains of emotional development (Laible and Thompson, 1998; Spangler and Zimmermann, 1999; Steele et al., 2008). It is especially influential for emotion regulation (Cassidy, 1994) as attachment patterns represent specific organizations of interactive emotion regulation (Zimmermann, 1999) that develop early in ontogeny. Secure attachment during childhood and adolescence is characterized by the ability to effectively regulate negative emotions with the caregiver (effective social emotion regulation). In addition, securely attached children and adolescents do regulate their emotions individually as well. In contrast, insecure-avoidantly attached children or adolescents try to regulate negative emotions individually without the caregiver in emotionally stressful situations. However, their regulation attempts often remain ineffective. Children and adolescents with insecure-ambivalent attachment organization typically show social but ineffective emotion regulation patterns for negative emotions with the caregiver as the contact and communication with the caregiver does not effectively reduce their negative emotions. The central characteristic of disorganized attachment is the absence or a breakdown of a coherent attachment strategy regulating emotional challenges (Main and Solomon, 1990; George and Solomon, 1999).

There is ample empirical evidence that attachment in childhood and adolescence is associated with emotion regulation (Kobak et al., 1993; Cassidy, 1994; Zimmermann, 1999; Waters et al., 2010; Brumariu, 2015; Zimmer-Gembeck et al., 2015) and even has effects on emotion related psychophysiology (Gander and Buchheim, 2015) also in adolescents’ interaction with their mothers (Spangler and Zimmermann, 2014).

Zimmermann et al. (2009) tested whether attachment and genetic variations of the 5-HTTLPR affect emotionality, emotion regulation, and aggression in early adolescence in a social talk show task eliciting social evaluative fear. They reported that S-allele-carriers of the 5-HTTLPR polymorphism were more aggressive and showed a higher emotional reactivity to restrictions of their autonomy. However, given secure concurrent attachment the S-allele- carriers of 5-HTTLPR showed more *agreeable autonomy* (i.e., assertion of own intentions with on-going communication with mother), whereas those with insecure attachment expressed more aggressive *hostile autonomy* (i.e., contradictions with verbal or physical aggression or emphasized refusal of further cooperation). Interestingly, there was no direct genetic or attachment effect on emotional expression, suggesting that eliciting negative emotions in adolescents works independently of attachment security and the serotonin transporter polymorphism. Thus, attachment and the 5-HTTLPR might be more influential on emotion regulation and hostile autonomy than on emotion expression, at least in a dominantly fear eliciting situation.

The current study tries to extend these results to a different situation that is dominantly eliciting anger. The main objective of the study was to investigate influences of adolescents’ genetic differences of the 5-HTTLPR and concurrent attachment on their emotional reactivity, emotion regulation, and aggressive hostile autonomy. In addition, we wanted to examine whether mother’s 5-HTTLPR explains her behavior towards the adolescent or explains the adolescent’s behavior towards her. Similarly, we wanted to test possible eliciting effects of the adolescents’ genotype on maternal behavior.

Similar to the study by Zimmermann et al. (2009), we expected no main effect of attachment and the 5-HTTLPR on emotionality as assessed in emotional expression. However, we expected that adolescents with both insecure attachment and the short S-allele would show more ineffective emotion regulation and aggressive hostile autonomy. Attachment security is expected to be a moderator of the genetic disposition associated with the 5-HTTLPR. This effect should be observed even when controlling for maternal intrusiveness as a concurrent environmental factor that differentially can elicit emotion dysregulation or hostile autonomy in the adolescent. In addition, we did expect that maternal 5-HTTLPR does not contribute to her intrusiveness or adolescents’ emotion related behavior.

## Procedures and Methods

### Participants

The current study was conducted as part of the 12-year longitudinal follow-up assessment of the Regensburg Longitudinal Study IV, a sample of originally 106 healthy German, Caucasian, low-risk infants (53 girls/53 boys), first assessed at 12 months of age. At the 12-year assessment, 96 early adolescents (49 girls/47 boys) and their mothers participated again in a series of tasks and interviews. According to maternal education assessed at follow-up, the families represent a wide range regarding their socioeconomic status, including 28% high school education (including university entrance certificate), 33% medium secondary school certificate, and 39% lower secondary education (most of them with additional vocational training).

The complete data set for this report was not available for all subjects due to missing values, which occurred because of technical problems with some video tapes, and in some cases due to the time schedule of families who had to leave before this task was completed. Therefore, specific statistical analyses include only a reduced sample size of *N* = 88.

### Procedures

At the age of 12, adolescents came to the university lab together with their mothers. After obtaining informed consent from the parents, the adolescents were interviewed regarding their attachment pattern to both parents. Afterwards, the adolescents and their mothers participated in two standardized interaction tasks designed to induce negative emotions. In this article, we report the results of the second interaction task, the dyadic computer game task. The Ethics Committee of the German Psychological Association has positively evaluated and accepted the design of the study.

#### Computer Game

The dyadic computer game is a modification of a task used in adolescent aggression research to induce anger in children and adolescents and to observe their aggressive behavior (de Castro et al., 2003). In the adaptation used here, mother and adolescent together played a computer-based jump and run game, with the aim to free a princess (“Esmeralda”) by controlling the play figure (“Quasimodo”) through several levels. The adolescent had two keys to control the movements of the play figure and the mother had one key. Both were instructed how to play the game and informed that they should carefully avoid pressing the “Ctrl-Key” (available on both sides of the keyboard, next to mother’s one key and next to the adolescent’s two keys), as this will lead to a game crash. After a short exercise phase, both mother and adolescent played a manipulated version of the game, where shortly before they successfully reached the final aim of freeing the princess, the game stopped with a sound announcing a game crash and presenting the sentence “The Ctrl-Key has been pressed” on the screen. A short time after the computer crash the experimenter entered the room and started the original program. The adolescents’ behavior and their mothers’ behavior were coded for emotion expression, emotion regulation, and hostile autonomy before and after the manipulated game crash. Independent and reliable coders carried out all behavior analyses using a standardized observational system.

### Measures

#### Twelve Year Measures of Emotion Expression, Emotion Regulation, and Hostile Autonomy

The analysis of the 12-year behavioral analysis included emotion expression, observed emotion regulation, and hostile autonomy of the adoelscent.

##### Emotion expression

Adolescents’ negative emotions were coded by means of a second-by-second event-based coding system from their facial and verbal expressions before and after the manipulated game crash. Negative emotions included anger, sadness, fear, and uneasiness. Inter-rater reliability was good (kappa 0.75).

##### Emotion regulation

Emotion regulation included those behaviors that modulate or change the emotional arousal of the adolescent. The coding separates the observed emotion expression from the observed emotion regulation process (Cole et al., 2004). Similar to the approach used by Buss and Goldsmith (1998) coders assessed strategy use and emotion expression after strategy use separately and rated the effectiveness of each strategy use. Each emotion regulation behavior was coded as either *effective* when the adolescent did no longer express the emotion after a time interval of maximum 7 s, or as *ineffective* if the duration was longer. Shorter emotion expressions were not coded regarding regulation effectiveness. Inter-rater reliability was good (kappa 0.72).

#### Adolescents’ Observed Hostile Autonomy

Autonomy represents the need for self-regulation achieved by being able to develop own goals or ideas for actions and striving to reach them. In the computer game task, autonomy was coded when the adolescent insisted on own ideas of playing the computer game or disagreed with his or her mother’s suggestions, commands or comments of what to do next. Disagreements with the mother were coded as *hostile autonomy* when they were followed or accompanied by verbal or physical attacks of the mother or by an explicit refusal to cooperate for at least 3 s. Events also were coded as hostile autonomy when the adolescent expressed emotionally charged responses towards the mother in response to an earlier disagreement with her. Examples of such attacks are active or reactive verbal threats, threatening gestures, physical attacks, or ridiculing utterances. Possible elicitors were the mothers’ tone of voice, her comments, or actions. Inter-rater reliability (kappa) was 0.84 for hostile autonomy.

#### Maternal Intrusiveness

Maternal intrusive behavior was defined as behavior inhibiting or undermining the adolescent’s autonomy and felt competence during the game. Typical maternal intrusive behaviors in the computer game include all actions interfering with the adolescent’s task activities, all derogating statements regarding the adolescent’s performance putting or pressure or achievement demands on the adolescent. Raters coded the number of intrusive maternal utterances. The inter-rater agreement resulted in a kappa of 0.78.

#### Adolescent Attachment Security

Attachment was assessed by the Late Childhood Attachment Interview (LCAI; Zimmermann and Scheuerer-Englisch, 2000), a semi-structured interview that probes the individual’s descriptions of the current relationship to both parents in attachment relevant situations and the attachment behavior towards the parents. The interviews were rated from videotapes with regard to attachment representations separately for attachment to mother and father and the adolescents’ attachment behavior when distressed and upset on 5-point scales. In addition, adolescents’ interview responses were coded regarding coherence with a categorical event-based system and regarding access to emotions. The adolescents’ attachment behavior scale and the adolescents’ attachment representations of mother scale were combined and utilized in the current study. Higher scores on this scale reflect seeking mother’s proximity, support or comfort when experiencing negative emotions that the adolescent cannot regulate without help, and coherently reporting maternal support and emotional availability. Low scores reflect avoidance of the mother in times of distress, retreat, or pretending that no help is needed as well as reports of maternal rejection, lack of emotional availability or inability to sooth or regulate the adolescent effectively. The interviews were rated by an independent coder who did not know other data of this study. Reliability (kappa), established on 20 interviews from a different sample, was 0.93.

The validity of the LCAI has been demonstrated in previous studies. The attachment classification in the strange situation in infancy and the parent-child interaction in toddlerhood is significantly associated with secure attachment behavior in the LCAI. In addition, concurrent parenting, and later attachment representations in adolescence assessed with the Adult Attachment Interview (AAI) is significantly associated with the attachment representation of mother in the LCAI (Zimmermann et al., 2000; Grossmann et al., 2002a,b).

#### Molecular-Genetic Analyses

Genotyping for the 5-HTTLPR polymorphism was performed at the Institute of Medical Chemistry, Molecular Biology and Pathobiochemistry, Semmelweis University (Budapest, Hungary), by scientists blind to the psychological data. Genomic DNA was isolated from buccal swabs using published procedures (Freeman et al., 1997).

The 5-HTTLPR variable number of tandem repeats (VNTR) polymorphism was investigated by employing two flanking primers for the polymerase chain reaction (sense primer: 5′ GGC GTT GCC GCT CTG AAT GC 3′, antisense primer: 5′ GAG GGA CTG AGC TGG ACA ACC AC 3′; thermocycling was initiated at 95°C for 10 min to activate HotStar DNA polymerase (Qiagen) followed by 35 cycles of 1 min denaturation at 95°C, 1 min of annealing at 65°C, and 1 min extension at 72°C, completed by 10 min of extension at 72°C. In both VNTR polymorphisms 50% of dGTP were replaced with dITP in order to avoid allelic drop-out in heterozygotes. The length of the generated PCR-amplicons directly reveals the repeat number (Ronai et al., 2001).

#### Task Duration

As mother-adolescent dyads differed somehow in the time they played the computer task, and in the time they spent discussing whose fault it was that the game crashed we also measured the task duration in seconds.

### Statistical Analyses

Two-factorial genotype × attachment quality analyses of variance were applied to test the hypotheses of this study. For use as independent factors, the 5-HTTLPR polymorphism and the attachment measures were dichotomized. Subjects were grouped into carriers and non-carriers of a short allele (ss and sl vs. ll) of the 5-HTTLPR, based on theoretical considerations and sample characteristics. Many scientific studies and meta-analysis compare carriers of at least one s-allele with ll-allele carriers (e.g., Munafò et al., 2008) and contrast them regarding amygdala reactivity to emotional stimuli and functional differences in serotonin regulation (Canli and Lesch, 2007). Moreover, the relatively low frequency of ss carriers (20%) in this study as reported in the next section, would have led to inappropriately small cell sizes for the planned two-factor gene × attachment analysis of variance (ANOVA) design. Similarly, subjects were grouped according to their scores on the combined attachment measure into subjects with insecure attachment (score < 3; which include avoidant and ambivalent attachment patterns) vs. secure (score > 3; representing secure attachment behavior and representation). Maternal intrusiveness and duration of the interaction task were included as covariates in the ANOVAs for autonomy, emotion expression, and observed emotion regulation to control for potential influences of maternal behavior and differences in gaming speed.

## Results

### Preliminary Analyses

The frequencies of the distribution of short and long allele variations of the 5-HTTLPR in the complete adolescent sample were 42% and 58%, respectively, which are comparable to the European population (Gelernter et al., 1997). The genotype frequencies were 35 (36%), 42 (44%), and 19 (20%) for the ll, ls, and ss genotypes, respectively. The 5-HTTLPR genotype distribution was in the Hardy-Weinberg equilibrium (χ^2^ (2, *N* = 96) = 0.96, ns). The genotype frequencies for the mothers in this sample were 31 (32%), 50 (52%), and 15 (16%) for the ll, ls and ss genotypes, respectively. The carriers of the ss and sl genotype were grouped together to avoid too small cell sizes for two-factor ANOVAs.

The number and duration of negative emotional expressions clearly show that the computer task did elicit negative emotions in the adolescents. The adolescents’ 5-HTTLPR-genotype status (ll vs. ss/sl) did not significantly differ with respect to maternal intrusiveness (*t*_(86)_ = −0.26, ns) or attachment security (*t*_(92)_ = 0.16, ns). However, adolescents with the long (ll) variant of the 5-HTTLPR-genotype were significantly faster (*M* = 149.3 s, SD = 50.2) in the computer game (*t*_(86)_ = −2.4, *p* = 0.024) compared to adolescents with at least one short (ss/sl) variant of the 5-HTTLPR-genotype (*M* = 180.0 s, SD = 66.7). Maternal 5-HTTLPR-genotype status (ll vs. ss/sl) did not significantly differ with respect to maternal intrusiveness (*t*_(86)_ = −1.5, ns) or adolescents’ attachment security (*t*_(92)_ = 0.15, ns) or duration of the computer game (*t*_(86)_ = 0.15, ns).

These results indicate that neither the mothers’ nor the adolescents’ 5-HTTLPR variations were significantly associated with maternal intrusiveness or the adolescent’s attachment security. In addition, there was no significant effect for adolescent’s gender regarding 5-HTTLPR-genotype status (χ^2^ (1, *N* = 96) = 0.63, ns), attachment security (*F*_(1,92)_ = 3.1, ns), or maternal intrusiveness (*F*_(1,86)_ = 0.08, ns). Moreover, gender was not significantly associated with adolescents’ hostile autonomy in the computer game (*F*_(1,86)_ = 0.09, ns), the frequency of their observed effective (*F*_(1,87)_ = 2.4, ns) or observed ineffective emotion regulation (*F*_(1,87)_ = 2.9, ns). However, girls more frequently expressed negative emotions than boys (*F*_(1,87)_ = 6.3, *p* = 0.014; (M_girls_ = 9.1; SD = 7.9; M_boys_ = 5.1, SD = 5.6) and also showed a longer duration of expression of negative emotions (*F*_(1,86)_ = 6.6, *p* = 0.012; M_girls_ = 26.0 s; SD = 20.7; M_boys_ = 13.3 s, SD = 16.5) but did not spend more time playing.

Maternal intrusiveness was significantly associated with adolescent’s hostile autonomy (*r*_(88)_ = 0.57, *p* < 0.01) and observed *ineffective* emotion regulation (*r*_(88)_ = 0.33, *p* < 0.01) but not with observed *effective* emotion regulation. For further statistical analyses, maternal intrusiveness, and duration of the computer game were included as covariates.

### Gene-Attachment Interactions

First, we tested a possible influence of genotype and attachment security on emotion expression in the dyadic interaction task. A 5-HTTLPR (ll vs. sl/ls) × attachment (insecure vs. secure) ANOVA with frequency and duration of negative emotional expression as dependent variables and maternal intrusiveness and task duration as covariates did not result in significant main or interaction effects. Thus, there was no significant gene or attachment effect on the adolescents’ emotional reactivity as observable in the negative emotional expressions (see Table 1).

**Table 1:** **Adolescents’ observed negative emotionality and emotion regulation (Means and SE)**.

	ll 5-HTTLPR		ss/sl 5-HTTLPR	
	Insecure attachment (*n* = 17)	Secure attachment (*n* = 17)	Insecure attachment (*n* = 28)	Secure attachment (*n* = 25)
**Negative emotionality**
Frequency	4.35 (0.84)	8.00 (1.99)	8.36 (1.49)	6.72 (1.27)
Duration (seconds)	11.83 (2.38)	21.56 (6.09)	25.15 (5.68)	21.05 (3.42)
**Emotion regulation**
Effective	2.18 (0.40)	3.35 (0.67)	2.46 (0.38)	3.12 (0.47)
Ineffective	2.29 (0.68)	3.29 (0.83)	4.82 (0.83)	2.88 (0.42)

Next, we examined the hypothesis of a possible gene-attachment interaction in explaining the adolescents’ observed emotion regulation. A 5-HTTLPR (ll vs. sl/ls) × attachment (insecure vs. secure) ANOVA with the frequency of effective observed emotion regulation as dependent variables and maternal intrusiveness and task duration as covariates did not result in significant main or interaction effects for observed emotion regulation. The same analysis for ineffective observed emotion regulation did not reveal significant main effects for 5-HTTLPR (*F*_(1,87)_ = 0.96, ns) and for attachment security (*F*_(1,87)_ = 0.93, ns). However, the gene × attachment interaction effect nearly reached significance (*F*_(1,87)_ = 3.9, *p* = 0.051). *Post hoc t*-tests revealed that adolescents with at least one short allele of the 5-HTTLPR who were in the secure attachment group showed significantly less observed *ineffective* emotion regulation (*t*_(39,8)_ = 2.1, *p* = 0.04) compared to adolescents with the short allele of the 5-HTTLPR from the insecure attachment group (see Table 1). There was no significant attachment effect on ineffective emotion regulation for the homozygote long allele carriers. However, given insecure attachment, ll-carriers showed less ineffective emotion regulation compared to ss/sl- carriers (*t*_(42,8)_ = 2.4, *p* = 0.023).

**Figure 1:**
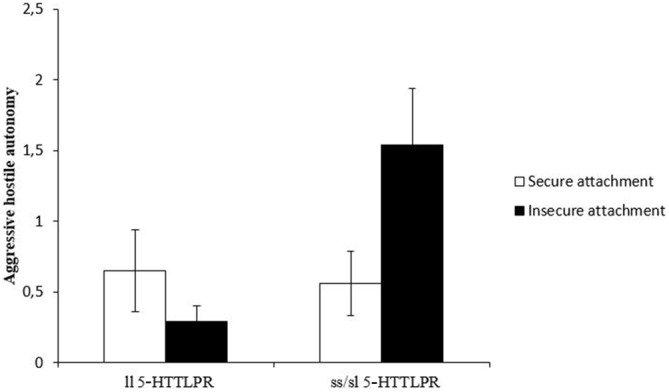
**Frequency (mean and SE) of adolescents’ aggressive hostile autonomy behaviors in the computer game task while interacting with their mothers**.

Finally, we examined the hypothesis of a possible gene-attachment interaction in explaining adolescents’ hostile autonomy behavior. A 5-HTTLPR × attachment ANOVA with hostile autonomy as dependent variables and maternal intrusiveness and task duration as covariates revealed a significant main effect for the 5-HTTLPR polymorphism (*F*_(1,86)_ = 4.5, *p* = 0.038), no significant main effect for attachment security (*F*_(1,86)_ = 0.53, ns) but a significant interaction effect (*F*_(1,87)_ = 5.2, *p* = 0.025) on hostile autonomy. As can be seen from Figure 1, the gene main effect indicates an increased frequency of hostile autonomy for short allele carriers of the 5-HTTLPR polymorphism. However, attachment security moderates this effect in two ways. Securely attached ss/sl carriers of the 5-HTTLPR polymorphism showed significantly less aggressive *hostile* autonomy (*t*_(42,7)_ = −2.1, *p* = 0.041) compared to insecurely attached adolescents with the short variant of the 5-HTTLPR (see Figure 1). In addition, within the group of insecurely attached adolescents, ss/sl carriers show significantly increased rates of aggressive *hostile* autonomy compared to ll-carriers (*t*_(31,2)_ = −3.0, *p* = 0.005). However, in the secure attachment group no significant 5-HTTLPR differences in hostile autonomy were found.

## Discussion

The primary goals of this study were to examine the interaction between molecular genetic polymorphisms of the serotonin transporter gene and attachment security in adolescence on emotionality, emotion regulation, and hostile autonomy as observed in an anger eliciting social context. Studies on the effects of the polymorphism in the promoter region of the serotonin transporter gene suggest an effect on emotional reactivity (i.e., amygdala activation) and attention to negative emotional stimuli and as a consequence, on emotion-regulation (Canli and Lesch, 2007; Canli et al., 2009; Hawn et al., 2015). However, surprisingly few genetic studies assess both emotionality and emotion regulation at the same time or include observable emotion regulation behavior as an outcome. As one of these few studies, Amstadter et al. (2012) showed that S-allele carriers gave up earlier during a frustrating task, an indirect hint for poor emotion regulation. However, the study did not report effects on emotionality. In this study, we successfully elicited negative emotions and coded emotional expression followed by the coding of effective or ineffective emotion regulation. Thus, the research is in line with suggestions for experimental emotion regulation research in developmental psychology (Cole et al., 2004). The task indeed did elicit negative emotions, which corroborates the internal validity of this task.

Replicating findings of a previous study (Zimmermann et al., 2009), neither the 5-HTTLPR polymorphism nor the attachment security predicted frequency or duration of negative emotional expression. Thus, the computer game task is comparably valid for all adolescents in eliciting negative emotionality when experiencing difficulties and failure in a dyadic computer game. In contrast to studies on amygdala activation, this study showed that the expressed negative emotionality seems to be independent of the serotonin transporter polymorphism. Oher studies report only more emotional expression in ss-carriers when combining positive and negative emotions (Gyurak et al., 2013). In addition, attachment security is associated with a wide range of open emotional expression, also of negative emotions, in contact with caregivers or with peers (Bretherton, 1990; Cassidy, 1994). Negative emotional expression would be restricted in insecure-avoidantly attached adolescents in interaction with the caregiver. However, as the computer task forces both adolescents and mothers to look at the screen and not to look at each other, display rules of insecurely attached adolescents might not influence emotional expression here. Similar attachment effects on emotion expression have been reported for adolescents’ interactions with close friends during a computer based task (Zimmermann et al., 2001).

The effects on emotional dysregulation (i.e., inefficient emotion regulation) showed a clear gene-attachment interaction. Carriers of the short SERT allele only showed increased emotional dysregulation when insecurely attached but not when securely attached. Thus, secure attachment seems to buffer adolescents against an impulsive genetic disposition for emotional dysregulation. Similarly, s-carriers of the 5-HTTLPR only showed increased aggressive *hostile* autonomy when insecurely attached but not when securely attached. Thus, secure attachment is a buffer against an impulsive genetic disposition for impulsive aggressive behavior.

The study revealed a heightened emotional dysregulation, especially to restrictions of autonomy for short 5-HTT polymorphism carriers in adolescence, an age period in which autonomy restrictions typically elicit negative emotions and contribute to quarrels with parents (Laursen, 1995). Carriers of the short 5-HTT polymorphism were significantly more sensitive to maternal comments and restrictions of their autonomy regarding how to play the game, what to do next, who is to blame after the computer crash compared to ll-carriers. This led to more impulsive contradictions, aggressive assertiveness or attacks of the adolescents against their mother but only in case of insecure attachment. In contrast, secure attachment seems to buffer against this genetic tendency to contradict maternal comments or commands. Secure internal working models might contribute to the adolescents’ expectations of trust towards their mother or an interpretation of her comments and commands as not undermining a general felt acceptance and security especially when emotionally challenged. The results also support the idea of differential susceptibility of adolescents who are carriers of the s-allele of the 5HTTLPR (Belsky, 2015). Within the group of ss/sl carriers, those with secure attachment did not show increased emotion dysregulation or aggressive hostile autonomy. The development of secure attachment in adolescence still depends on emotional available caregiving and support (Zimmermann, 1999; Allen and Land, 2008). Thus, short allele carriers seem to be more sensitive to effects of attachment security based on their attachment relevant caregiving experience. However, the results even speak more for a transactional developmental pattern. Differential susceptibility does not subsume the idea of an active individual. As attachment is a stable characteristic of the adolescent, the observed emotion dysregulation, and aggressive hostile autonomy is not only reactivity or sensitivity to the concomitant maternal comments as concurrent environment. Adolescent attachment moderates the responses the current environment and the genetic tendency associated with the s-allele.

Thus, at least in adolescence, the 5-HTTLPR does not seem to affect emotionality as observable in an increased frequency or duration of negative emotional expression but particularly in aggressive reactions to stage-salient emotional elicitors of negative emotions, such as restrictions of autonomy in adolescence (Laursen, 1995). The participants of this study were in early adolescence, an age with a lowered repertoire of emotion regulation strategies (Zimmermann and Iwanski, 2014). The many conflicts with parents at that age (Laursen et al., 1998) may increase the allostatic load of adolescents with the combination of the 5-HTTLPR S-allele and insecure attachment by a heightened physiological stress response (Cook et al., 2015) starting a maladaptive cascade which leads to an increased risk for psychopathology (Masten and Cicchetti, 2010). There is growing evidence 5-HTTLPR—environment interactions on the development of externalizing behavior in the last years (Brett et al., 2015; Cline et al., 2015).

Beside the effects on adolescent behavior the study also showed that mothers’ 5-HTTLPR was not associated with her intrusive behavior towards the adolescent. In concordance with Bakermans-Kranenburg and van IJzendoorn (2008), mothers who are ss/sl carriers are not more intrusive, which suggests that we do not have the evidence of a specific genetic effect on mothers caregiving in this situation associated with the short allele of 5-HTTLPR. In addition, also adolescents who are ss/sl carriers did not significantly elicit more intrusive behavior in their mothers. Thus, we have not evidence of a genetic eliciting effect of the adolescent on this specific parenting behavior in this task. This further supports the idea that the moderation of adolescents’ genotype (5-HTTLPR) by attachment is a sign of transactional processes in adolescence where attachment already is a characteristic of the person that influences the effect of genetic dispositions independent of the concurrent maternal caregiving environment.

Clearly, there are methodological limitations of this study. The sample size may have been too small to have enough power to detect more direct or interaction effects (Murphy and Lesch, 2008). As this was a longitudinal follow-up study, the sample size was somehow restricted which decreases statistical power. Extended replications with other age groups and in interaction with fathers or peers are required. In addition, the effects of other cumulative environmental risks (Caspi et al., 2003) or the interaction with other candidate genes clearly need to be considered. Moreover, from a transactional developmental perspective the direction of the effects might be more complex than has been studied here. Longitudinally, impulsive individuals also can elicit more negative evaluations and expectations in their social environment, which in turn increase the emotional dysregulation observed during interactions.

However, despite these limitation the study adds new evidence that attachment can moderate the effect of the short variant of the 5-HTTLPR on emotional dysregulation and impulsive aggression.

## Author Contributions

PZ and GS contributed to the design of the study, the data analysis, and the manuscript.

## Funding

This research has been supported by the Koehler-Stiftung (Munich, Germany) and the German Research Foundation (SP 312/16-1 and ZI 511/13-1). We are very indebted to Fatma Çelik for behavior codings and Szolt Ronai for the genetic analysis. Finally, we want to underline our gratitude to the families for their extraordinary cooperation.

## Conflict of Interest Statement

The authors declare that the research was conducted in the absence of any commercial or financial relationships that could be construed as a potential conflict of interest.
